# Effect of Physical Activity on Health-Related Quality of Life of Older Adults Using Newly Developed Health-Related Quality of Life Tool for the Korean Population

**DOI:** 10.3390/healthcare11152192

**Published:** 2023-08-03

**Authors:** Jungwoon Seo, Sangyong An, Daehee Kim

**Affiliations:** 1 Department of Physical Education, Dongguk University, Seoul 06420, Republic of Korea; dongguk820@gmail.com (J.S.); ansang0711@gmail.com (S.A.); 2 College of Information Technology and Convergence, Pukyong National University, Busan 48513, Republic of Korea

**Keywords:** physical activity, health-related quality of life, older adults

## Abstract

This study aimed to investigate the effect of physical activity on health-related quality of life. Data from the 2019 Korean National Health and Nutrition Examination Survey were used. The participants were 1495 (56.7% women) older individuals older than 65 years of age. A one-way analysis of variance (ANOVA) and multiple regression analysis were performed to determine the effect of walking and strength exercise on the health-related quality of life using the Korean version of the health-related quality of life questionnaire called the Korean Health-Related Quality of Life Instrument with 8 Items (HINT-8). The one-way ANOVA revealed that the health-related quality of life (according to the HINT-8) differed depending on increases in walking and strength exercise. Furthermore, walking and strength exercise positively influenced the health-related quality of life according to the HINT-8. Therefore, this study showed that walking and strength exercise were associated with the health-related quality of life among older Korean individuals. This study will be helpful for future studies on the health-related quality of life of older individuals.

## 1. Introduction

The population comprising individuals older than 65 years of age has been remarkably growing for years and receiving copious attention. The United Nations highlighted that in 2050, 1 in 6 individuals worldwide will be older than age 65 years (16%), which is an increase from 1 in 11 (9%) in 2019. Furthermore, the number of individuals older than age 65 years is predicted to double between 2019 and 2050 in several countries [1]. The United Nations and the World Health Organization have divided older individuals into three groups: more than 7% of the total population is an “aging society”; more than 14% of the total population is an “aged society”; and more than 20% of the total population is a “super-aged society” [2]. This explains the population of people aged > 65 years. 

Korea regards those older than 65 years of age as older individuals [3,4]. The results of a survey of the Korean population have shown that the proportion of individuals at least 65 years of age has reached 16.5%, thus comprising an aged society; this implies that Korea will soon encompass the next level of the aging society, with this proportion expected to reach 20.3% in 2025 and 43.9% in 2060, thus becoming a super-aged society [5].

Because of the deterioration of mental health and a decline in physical ability and dementia, older individuals in an aged society experience deprivation of their social presence, resulting in a sense of loss; these factors negatively affect the health-related quality of life (HRQoL) by creating a feeling of social isolation [3,4]. Moreover, the importance of physical activities is emphasized for the maintenance or improvement of mental health and physical health because they can prevent degradation factors associated with HRQoL, such as psychological problems and the decreased capacity to exercise [6]. Moreover, because conducting physical activities prevents and protects against decreased physiological functioning which is inevitable for older people [7], physical activities and exercise are significant for improving quality of life from a physiological perspective [8].

Physical activities and the HRQoL of older individuals are interesting issues that have been continuously studied [9,10,11,12,13]. Furthermore, functions and attributes related to HRQoL include physical, mental, and social functions that are associated with sustaining the ability to feel relaxed or happy, to perform physical, mental, and intellectual functions, and to maintain a satisfactory perception of social activity and valuable health attributes [14]. Society not only concentrates on the health of older individuals but also focuses attention on and agonizes about improving the HRQoL of older individuals to maintain their health during the golden years [15]. Therefore, in modern society, which includes living in the age of aging, the subjects of physical activity, health, and quality of life of older adults have been emerging as social concerns.

Physical activity is a very effective way to improve the quality of life as well as physical health and mental health [16,17]. Park [18] claimed that aging results in decreased muscle strength or physical strength and physical function, thus negatively affecting the quality of life. An et al. [19] argued that improvements in physical health and quality of life are positively influenced by strength exercise. Strength exercise is an essential workout that improves the quality of life of older adults [20]. Physical activity exists in various forms of exercise for older people. Considering the characteristics of older people, it is suggested that walking and strength exercise, such as push-ups or sit-ups, which are without relative time, place, and cost problems, are less burdensome and effective for these individuals. Walking exercise does not need special education and training and is possible for everyone to perform; furthermore, it is not limited by age and is easy to practice, its intensity can be controlled, and it involves a low risk of injuries [9,12,13]. Accordingly, such advantages prove that walking exercise is considered an appropriate exercise for older individuals [21,22].

For several years, the European Quality of Life 5 Dimensions (EQ-5D) questionnaire has been used to measure the HRQoL through the Korea National Health and Nutrition Examination Survey (KNHANES), which has been used since 1998 to provide information on Korean health and nutritional status to establish national health policy in Korea; however, the Korea Centers for Disease Control and Prevention created a Korean version of the HRQoL questionnaire called the Korean Health-Related Quality of Life Instrument with 8 Items (HINT-8) that includes climbing stairs, pain, vitality, working, depression, memory, sleep, and happiness; furthermore, it comprises a four-point Likert-type scale for assessment [23]. The HRQoL of Korean individuals has been measured by instruments such as EQ-5D that were not completely suitable for the Korean population. Therefore, the HINT-8, which was specifically created for Korean individuals, is a highly appropriate and meaningful tool for measuring HRQoL in this population. However, the HINT-8 was first used in 2019, and studies based on this tool are insufficient [12,13]. Furthermore, studies evaluating walking and strength exercise variables, together with the HINT-8 outcomes of older individuals, have not been performed. Therefore, such studies are necessary [9,10,12,13,24].

This study aimed to prove the HINT-8 results of older individuals who perform walking and strength exercises and analyze how physical activities influence the quality of life of older individuals. Moreover, this study supports the connection between the importance of social presence, physical activities, and quality of life of older individuals. We believe that this study can be used as a basis for future research on the influence of physical activities on older people and the improvement of older people’s quality of life.

## 2. Materials and Methods

This study used raw data from the 8th National Health and Nutrition Survey (2019) based on Article 16 of the National Health Promotion Act, which involves government-designated statistics (approval number 117002), and Article 17 of the Statistical Law.

The National Health and Nutrition Survey was performed every 3 years from 1998 to 2008. However, after 2008, it has been conducted every year. Approval was obtained from the Korea Disease Control and Prevention Agency institutional review board to use announcements, distributed press releases, published statistics, and raw data. This study was approved by the institutional review board (2018-01-03-C-A) for the collection of human biologic material and provision of raw data from a third party.

### 2.1. Measures

#### 2.1.1. Physical Activity

Data were obtained from the KNHANES website and the Korean version of the Global Physical Activity Questionnaire, which has verified credibility and validity, was used. The Global Physical Activity Questionnaire comprises questions about performing physical activities during 1 week. It was created by the World Health Organization and is a standardized tool used to measure the levels of physical activities such as work, leisure, and travel [25]. Because participants were aged > 65 years of age, they universally responded that they perform physical activities during daily life. This study divided physical activity into walking and strength exercises and defined how often participants performed these during 1 week. The participants assessed their frequency of walking and strength exercise during 1 week using a 4-point Likert scale (1 = not at all; 2 = 1–2 days/week; 3 = 3~4 days/week; 4 = more than 5 days/week).

#### 2.1.2. Korean Health-Related Quality of Life Instrument with 8 Items

The HINT-8 was created by the Korea Centers for Disease Control and Prevention in 2014, and it has been used with the KNHANES [26]. The HINT-8 includes eight items, climbing stairs, pain, vitality, working, depression, memory, sleep, and happiness, which are rated using a 4-point Likert scale (1 = always good; 2 = usually good; 3 = sometimes good; 4 = poor). Lower scores indicate better HRQoL (1 = I am always happy; 2 = I am usually happy; 3 = I am sometimes happy; and 4 = I am never happy).

#### 2.1.3. Data Analysis

In this study, we used KNHANES data which are weighted in consideration of the extraction rate, response rate, and population distribution of the subjects selected by the stratified sampling method, and measurement errors and processing errors were corrected and supplemented in the first collected original data. In order for this data to represent the entire population of Korea, the weight was reflected in the data analysis according to the guidelines for using raw data presented by the National Health and Nutrition Survey. Therefore, we calculated the weighted values of the health examination survey (wt_ivex), the variance estimate layer (kstrata), and the number of enumeration districts (psu) to conduct a complex sampling analysis because the KNHANES data were created as a stratified sampling model. After determining the general characteristics of the study participants older than 65 years of age (sex, education level, marital status, economic activity) and their physical activity practices (walking and strength exercise), a frequency analysis was performed using SPSS version 26. The Kolmogorov–Smirnov test was performed to verify normal distribution. Continuous variables are presented as mean and standard deviations. A one-way analysis of variance (ANOVA) was applied to understand the differences in HINT-8 results in accordance with physical activities. Then, the Duncan test, as a post hoc test, was performed. The correlation between walking exercise, strength exercise, and HINT-8 results was examined by Pearson’s product–moment correlation. Moreover, we implemented a multiple regression analysis to statistically verify the effects of walking and strength exercise on the HINT-8 results. Using the G power analysis 3.1, the necessary sample size for the one-way ANOVA was estimated to be 252 and that for the multiple regression analysis was 172 [27,28]. Finally, a significance (two-tailed) level of *p* < 0.05 was set for all the analyses during this study.

## 3. Results

To analyze the general characteristics of study participants, we first separated KNHANES data of 8110 individuals into those <65 years and those >65 years. As a result, we secured the data of 1735 individuals >65 years of age. Finally, we extracted the data of 1495 individuals (56.7% women and 43.3% men) by eliminating data of those <65 years, nonresponses, and improper data. The proportions of participants based on the level of education were as follows: (1) less than elementary school: 54.6%; (2) graduated from high school: 19%; (3) graduated from middle school: 16.5%; and 4) university degree or higher: 9.9%. The proportion of participants based on marital status was as follows: (1) married: 67.1%; (2) widowed: 27.6%; (3) divorced: 4%; (5) and separated: 1.3%. Regarding their economic activity, 64.8% had no economic activity while 35.2% had economic activity. The frequency rates of performing walking exercise were as follows: (1) more than five days/week: 43.3%; (2) not at all: 23.4%; (3) three or four days/week: 20.1%; and (4) one or two days/week: 13.2%. The frequency rates of performing strength exercise were as follows: 1) not at all: 81.1%; (2) more than five days/week: 10%; (3) three or four days/week: 5.6%; and (4) one or two days/week: 3.3%. The characteristics of the study participants are provided in Table 1.

The one-way ANOVA results indicated that walking and strength exercise had significant effects on the HINT-8 results, except those for memory related to strength exercise. As mentioned, lower HINT-8 scores indicated positive responses during this study. The outcome of the ANOVA revealed that the means and standard deviations of the HINT-8 scores were statistically changed by the frequency (days) of physical activity performance (Table 2 and Table 3).

The correlation analysis results (Table 4) indicated significant relationships among the measured variables. Although the findings revealed a negative relationship between physical activities and HINT-8 scores, there was a positive relationship between all HINT-8 variables.

We conducted a multiple regression analysis to further explain the effects of physical activities on HINT-8 scores (Table 5). Walking and strength exercise negatively affected HINT-8 scores, which means they positively affected all HINT-8 variables.

## 4. Discussion

During this study, we explored the effects of walking and strength exercise on the results of the HINT-8, a newly developed HRQoL tool for the Korean population, among individuals older than 65 years. The HINT-8 results showed differences in satisfaction among individuals that were dependent on the frequency of walking and strength exer cise and that physical activity performance statistically influenced the HINT-8 scores.

The differences in HINT-8 scores dependent on the frequency of physical activity performed by those older than 65 years of age showed that vitality and happiness were influenced by walking exercise three or four days/week and that climbing stairs, vitality, working, depression, and happiness were influenced by strength exercise three or four days/week, resulting in HINT-8 scores indicating the highest satisfaction. Moreover, other physical activity variables were associated with higher satisfaction when the frequency of activity was higher. Therefore, the HINT-8 scores showed that increased exercise frequency increased the satisfaction of the individuals; these results were similar to those of previous studies [9,22,24].

The comparison between the physical activities (walking and strength exercise) and HINT-8 results revealed statistically significant correlations between all variables. This means that the relationship between exercise and HINT-8 scores regarding the HRQoL will receive more attention and be studied more among older individuals to improve the health of the super-aged society.

Moreover, the findings of the effects of walking and strength exercise on the HINT-8 results supported our assumptions, verifying the positive effect of physical activity on HRQoL [29]. Walking exercise influenced climbing stairs, working, depression, memory, sleep, and happiness more than strength exercise. However, pain and vitality were more affected by strength exercise than walking exercise. Walking exercise positively influenced mental factors such as stress and depression, improved the ability to perform activities, and was associated with a low risk of injuries, thereby helping to increase the quality of life [10,30]. Furthermore, strength exercise had positive effects on the quality of life, and decreased strength was highly related to a poorer quality of life; these effects were similar to those found by other research studies [19,31].

This study aimed to determine how physical activities affect the HRQoL of individuals >65 years. Life extension, health, and quality of life are issues faced by our aging society; therefore, they are of great interest. This increased attention will positively affect the physical activities of older individuals, improving the HRQoL of the next generation. Other studies related to this topic have been initiated not only in Korea but also in many countries, including the United States, the United Kingdom, Sweden, Portugal, Singapore, and Taiwan [29,32,33,34,35,36,37]. This indicates that the importance of HRQoL is being emphasized. Furthermore, physical activities increase health, self-esteem, and life satisfaction and decrease mental diseases in older individuals, resulting in a high level of satisfaction in terms of the quality of life [33,38]. Physical activities positively affect a broad range of physiological systems [39]. For instance, physical activities for older people improve physiological points including the control of arterial blood pressure and vital organ perfusion, augmentation of oxygen and substrate delivery and utilization within the active muscle, maintenance of arterial blood homeostasis, and dissipation of heat, similar to those of young adults [40]. The growth of the population of older individuals has led to increased medical expenses, which account for 43% of their total medical costs [12,13]. The medical costs of older individuals are two times to five times higher than those of younger generations; therefore, medical expenses have become an important social issue with negative implications such as bankruptcy. However, these economic problems could be prevented or reduced by increased physical activity, improving physical functions that protect health [35]. Therefore, physical activities positively affect the maintenance of physical health and psychological health and improve the HRQoL as well as the medical expenses of older individuals.

The EQ-5D has been widely used to measure the quality of life. However, this study considered Korean characteristics to analyze the quality of life of Korean individuals older than 65 years of age by using the HINT-8. Because the HINT-8 was introduced for use with the KNHANES in 2019, few studies have investigated it. Therefore, this study is academically meaningful because the information provided can be used in future studies on this topic.

Although we found that physical activities have positive effects on HINT-8 scores, this study had some limitations. First, because the National Health and Nutrition Survey was performed, some data, such as the distributions of age groups and exercise levels, could not be extracted. Second, we could not obtain all data of those >65 years of age. Therefore, if more data on those >65 years of age are gathered, then the spectrum of future studies will become wider and more meaningful. Next, because sex, level of education, marital status, and economic activity could also be also great motivators for conducting physical activity, such variables should be considered in the next study. This could increase the level of R2 values in the regression analysis because such variables can positively affect the HINT-8. Finally, the HINT-8 was created only to be utilized in Korea; therefore, it is difficult to guarantee the reliability of the observed effects for individuals in other counties.

## 5. Conclusions

This study has verified that physical activities improve the HRQoL of Korean older people. The enhanced HRQoL could positively affect Korean older adults’ lives. By promoting physical activities, various social issues such as medical, financial, and political issues could be ameliorated, and resources and costs could be saved for older adults’ health. Every year, a significant amount of money is spent on older people’s healthcare. Therefore, encouraging physical activities, such as walking and other exercises, not only enhances their health but also enables them to contribute to society by working and being active members. Therefore, improving the HRQoL through physical activities could foster social development and national power. Finally, we aspire that the HINT-8 measurement tools can be used for older Korean people to prevent diverse risks in their lives and to improve their health and quality of life.

## Figures and Tables

**Table 1:** General characteristics of the study participants (*n* = 1495).

Characteristic		Frequency (*n*)	Percentage (%)
Sex	Male	647	43.3
	Female	848	56.7
Level of education	Less than elementary school	816	54.6
	Graduation from middle school	247	16.5
	Graduation from high school	284	19.0
	University degree or higher	148	9.9
Marital status	Married	1003	67.1
	Separated	19	1.3
	Widowed	413	27.6
	Divorced	60	4.0
Economic activity	Yes	526	35.2
	No	969	64.8
Walking exercise	Not at all	350	23.4
	1–2 days/week	197	13.2
	3–4 days/week	300	20.1
	More than 5 days/week	648	43.3
Strength exercise	Not at all	1212	81.1
	1–2 days/week	50	3.3
	3–4 days/week	84	5.6
	More than 5 days/week	149	10.0

**Table 2:** Differences in HINT-8 scores according to walking exercise frequency.

Variable	Frequency	Mean (SD)	F(t)	Duncan
Climbing stairs	Not at all ^c^	2.24 (0.858)	30.658 ***	a > b > c
	1–2 days/week ^b^	1.95 (0.766)		
	3–4 days/week ^a^	1.82 (0.694)		
	More than 5 days/week ^a^	1.78 (0.751)		
Pain	Not at all ^c^	2.00 (0.823)	10.314 ***	a > b > c
	1–2 days/week ^b^	1.87 (0.744)		
	3–4 days/week ^ab^	1.77 (0.648)		
	More than 5 days/week ^a^	1.74 (0.728)		
Vitality	Not at all ^c^	2.59 (1.107)	22.538 ***	a > b > c
	1–2 days/week ^b^	2.38 (1.036)		
	3–4 days/week ^a^	2.04 (0.977)		
	More than 5 days/week ^a^	2.11 (0.994)		
Working	Not at all ^c^	2.23 (0.998)	32.306 ***	a > b > c
	1–2 days/week ^b^	1.85 (0.837)		
	3–4 days/week ^ab^	1.73 (0.776)		
	More than 5 days/week ^a^	1.70 (0.799)		
Depression	Not at all ^b^	1.64 (0.840)	7.860 ***	a > b
	1–2 days/week ^b^	1.61 (0.743)		
	3–4 days/week ^a^	1.44 (0.600)		
	More than 5 days/week ^a^	1.45 (0.653)		
Memory	Not at all ^b^	1.90 (0.721)	6.489 ***	a > b
	1–2 days/week ^a^	1.79 (0.615)		
	3–4 days/week ^a^	1.78 (0.593)		
	More than 5 days/week ^a^	1.72 (0.615)		
Sleep	Not at all ^c^	1.88 (0.861)	7.887 ***	a > b > c
	1–2 days/week ^bc^	1.79 (0.755)		
	3–4 days/week ^ab^	1.68 (0.712)		
	More than 5 days/week ^a^	1.65 (0.703)		
Happiness	Not at all ^b^	2.55 (1.072)	12.406 ***	a > b
	1–2 days/week ^b^	2.50 (1.028)		
	3–4 days/week ^a^	2.19 (0.953)		
	More than 5 days/week ^a^	2.26 (0.970)		

HINT-8, Korean Health-Related Quality of Life Instrument with 8 Items; SD, standard deviation. *** *p* < 0.001, smallercase letters mean they are divided into groups in Duncan which is one of the methods from post hoc.

**Table 3:** Differences in HINT-8 scores according to strength exercise frequency.

Variable	Frequency	Mean (SD)	F(t)	Duncan
Climbing stairs	Not at all ^b^	1.99 (0.797)	17.321 ***	a > b
	1–2 days/week ^a^	1.70 (0.707)		
	3–4 days/week ^a^	1.59 (0.639)		
	More than 5 days/week ^a^	1.62 (0.709)		
Pain	Not at all ^b^	1.87 (0.754)	8.463 ***	a > b
	1–2 days/week ^ab^	1.66 (0.745)		
	3–4 days/week ^ab^	1.69 (0.652)		
	More than 5 days/week ^a^	1.58 (0.666)		
Vitality	Not at all ^b^	2.34 (1.049)	20.284 ***	a > b
	1–2 days/week ^a^	1.92 (0.876)		
	3–4 days/week ^a^	1.79 (0.921)		
	More than 5 days/week ^a^	1.81 (0.926)		
Working	Not at all ^b^	1.91 (0.892)	12.239 ***	a > b
	1–2 days/week ^a^	1.50 (0.762)		
	3–4 days/week ^a^	1.53 (0.680)		
	More than 5 days/week ^a^	1.62 (0.771)		
Depression	Not at all	1.54 (0.722)	3.320 *	-
	1–2 days/week	1.46 (0.542)		
	3–4 days/week	1.34 (0.589)		
	More than 5 days/week	1.41 (0.686)		
Memory	Not at all	1.80 (0.657)	1.877	-
	1–2 days/week	1.70 (0.580)		
	3–4 days/week	1.69 (0.486)		
	More than 5 days/week	1.71 (0.593)		
Sleep	Not at all	1.76 (0.779)	4.031 **	-
	1–2 days/week	1.68 (0.620)		
	3–4 days/week	1.63 (0.667)		
	More than 5 days/week	1.55 (0.628)		
Happiness	Not at all ^b^	2.40 (1.026)	5.755 ***	a > b
	1–2 days/week ^a^	2.10 (0.931)		
	3–4 days/week ^a^	2.08 (0.843)		
	More than 5 days/week ^ab^	2.17 (0.938)		

HINT-8, Korean Health-Related Quality of Life Instrument with 8 Items; SD, standard deviation. * *p* < 0.05, ** *p* < 0.01, *** *p* < 0.001, smallercase letters mean they are divided into groups in Duncan which is one of the methods from post hoc.

**Table 4:** Correlation analysis of physical activities and HINT-8 scores.

Variable	Walking Exercise	Strength Exercise	Climbing Stairs	Pain	Vitality	Working	Depression	Memory	Sleep	Happiness
Walking exercise	1									
Strength exercise	0.174 ***	1								
Climbing stairs	−0.240 ***	−0.178 ***	1							
Pain	−146 ***	−0.132 ***	0.474 ***	1						
Vitality	−0.183 ***	−0.189 ***	0.398 ***	0.325 ***	1					
Working	−0.227 ***	−0.133 ***	0.499 ***	0.403 ***	0.464 ***	1				
Depression	−0.113 ***	−0.079 **	0.298 ***	0.310 ***	0.368 ***	0.383 ***	1			
Memory	−0.112 ***	−0.058 *	0.285 ***	0.287 ***	0.295 ***	0.333 ***	0.321 ***	1		
Sleep	−0.118 ***	−0.087 **	0.275 ***	0.313 ***	0.289 ***	0.311 ***	0.373 ***	0.321 ***	1	
Happiness	−0.138 ***	−0.093 ***	0.251 ***	0.227 ***	0.371 ***	0.314 ***	0.458 ***	0.295 ***	0.357 ***	1

HINT−8, Korean Health-Related Quality of Life Instrument with 8 Items. * *p* < 0.05, ** *p* < 0.01, *** *p* < 0.001.

**Table 5:** Multiple regression analysis of significant factors associated with HINT-8 scores.

HINT-8 Domains	Variables	Unstandardized Coefficients		β	t(*p*)
		B	SE		
Climbing stairs	Constant	2.480	0.054		45.843 ***
	Walking exercise	−0.139	0.016	−0.213	−8.555 ***
	Strength exercise	−0.123	0.020	−0.157	−6.286 ***
	R = 0.285, R^2^ = 0.080, F = 67.215 ***, Durbin–Watson = 1.891				
Pain	Constant	2.138	0.053		41.585 ***
	Walking exercise	−0.072	0.016	−0.117	−4.572 ***
	Strength exercise	−0.089	0.019	−0.120	−4.685 ***
	R = 0.181, R^2^ = 0.033, F = 25.723 ***, Durbin–Watson = 1.901				
Vitality	Constant	2.873	0.073		39.166 ***
	Walking exercise	−0.136	0.022	−0.156	−6.194 ***
	Strength exercise	−0.181	0.027	−0.172	−6.810 ***
	R = 0.251, R^2^ = 0.063, F = 50.849 ***, Durbin–Watson = 1.960				
Working	Constant	2.377	0.059		40.101 ***
	Walking exercise	−0.151	0.018	−0.214	−8.492 ***
	Strength exercise	−0.087	0.022	−0.102	−4.060 ***
	R = 0.252, R^2^ = 0.064, F = 51.503 ***, Durbin–Watson = 1.864				
Depression	Constant	1.740	0.050		34.996 ***
	Walking exercise	−0.070	0.015	−0.120	−4.666 ***
	Strength exercise	−0.035	0.018	−0.050	−1.951
	R = 0.138, R^2^ = 0.019, F = 14.722 ***, Durbin–Watson = 1.942				
Memory	Constant	1.971	0.046		42.746 ***
	Walking exercise	−0.059	0.014	−0.110	−4.273 ***
	Strength exercise	−0.020	0.017	−0.031	−1.183
	R = 0.119, R^2^ = 0.014, F = 10.982 ***, Durbin–Watson = 1.994				
Sleep	Constant	1.990	0.054		37.082 ***
	Walking exercise	−0.071	0.016	−0.113	−4.405 **
	Strength exercise	−0.053	0.019	−0.070	−2.723 **
	R = 0.143, R^2^ = 0.020, F = 54.860 ***, Durbin–Watson = 1.914				
Happiness	Constant	2.777	0.072		38.693 ***
	Walking exercise	−0.115	0.022	−0.137	−5.320 ***
	Strength exercise	−0.080	0.026	−0.079	−3.064 **
	R = 0.169, R^2^ = 0.029, F = 22.181 ***, Durbin–Watson = 2.031				

HINT-8, Korean Health-Related Quality of Life Instrument with 8 Items; SE, standard error. ** *p* < 0.01, *** *p* < 0.001.

## Data Availability

Publicly available datasets were analyzed in this study. This data can be found here: https://knhanes.kdca.go.kr/knhanes/sub03/sub03_02_05.do (accessed on 25 May 2022).
